# Integrative network analysis of TCGA data for ovarian cancer

**DOI:** 10.1186/s12918-014-0136-9

**Published:** 2014-12-31

**Authors:** Qingyang Zhang, Joanna E Burdette, Ji-Ping Wang

**Affiliations:** Department of Statistics, Northwestern University, Evanston, IL60208 USA; Department of Medicinal Chemistry and Pharmacognosy, University of Illinois, Chicago, 60607 IL USA

**Keywords:** The Cancer Genome Atlas, Bayesian network, Pathway analysis, Feature selection, Causal inference, Directed network

## Abstract

**Background:**

Over the past years, tremendous efforts have been made to elucidate the molecular basis of the initiation and progression of ovarian cancer. However, most existing studies have been focused on individual genes or a single type of data, which may lack the power to detect the complex mechanisms of cancer formation by overlooking the interactions of different genetic and epigenetic factors.

**Results:**

We propose an integrative framework to identify genetic and epigenetic features related to ovarian cancer and to quantify the causal relationships among these features using a probabilistic graphical model based on the Cancer Genome Atlas (TCGA) data. In the feature selection, we first defined a set of seed genes by including 48 candidate tumor suppressors or oncogenes and an additional 20 ovarian cancer related genes reported in the literature. The seed genes were then fed into a stepwise correlation-based selector to identify 271 additional features including 177 genes, 82 copy number variation sites, 11 methylation sites and 1 somatic mutation (at gene *TP53*). We built a Bayesian network model with a logit link function to quantify the causal relationships among these features and discovered a set of 13 hub genes including *ARID1A*, *C19orf53*, *CSKN2A1* and *COL5A2*. The directed graph revealed many potential genetic pathways, some of which confirmed the existing results in the literature. Clustering analysis further suggested four gene clusters, three of which correspond to well-defined cellular processes including cell division, tumor invasion and mitochondrial system. In addition, two genes related to glycoprotein synthesis, *PSG11* and *GALNT10*, were found highly predictive for the overall survival time of ovarian cancer patients.

**Conclusions:**

The proposed framework is effective in identifying possible important genetic and epigenetic features that are related to complex cancer diseases. The constructed Bayesian network has identified some new genetic/epigenetic pathways, which may shed new light into the molecular mechanisms of ovarian cancer.

**Electronic supplementary material:**

The online version of this article (doi:10.1186/s12918-014-0136-9) contains supplementary material, which is available to authorized users.

## Background

Ovarian cancer, one of the most malignant gynecologic cancers, is the fifth leading cause of cancer-related deaths among women in the United States. According to the American Cancer Society, 21,980 women will receive a new diagnosis and 14,270 will die of this disease in 2014. The majority of ovarian cancers are serous ovarian carcinomas and only less than 20*%* of them can be detected early. High-stage cancer patients are usually treated with platinum/taxane-based chemotherapy after debulking surgery. Platinumresistant cancer recurs in approximately 25*%* of patients within six months after therapy, and the overall five-year survival probability is only 31% [1]. While the molecular mechanism of ovarian cancer remains unclear, studies have suggested that many different factors may contribute to this disease, among which there are tens of well-known oncogenes and tumor suppressors including *TP53*, *PIK3C*, *BRCA1* and *BRCA2*. In particular the mutation of gene *TP53* is the most common, occurring in at least 70*%* of advanced-stage cases [1,2]. Many of the existing studies however, have been focused on a single type of data, most frequently, gene expression analysis [3-5]. As pointed out by many researchers, the analysis based on individual gene often fail to provide even moderate prediction accuracy of the cancer status. Thus a systems biology approach that combines multiple genetic and epigenetic profiles for an integrative analysis provides a new direction to study the regulatory network associated with ovarian cancer.

The rapid advances in next-generation sequencing technology now allow genome-wide analysis of genetic and epigenetic features simultaneously. The timely advent of TCGA project has provided the most comprehensive genomic data resource from over 20 types of cancers (http://cancergenome.nih.gov/). For example, the TCGA ovarian cancer data contain both clinical and molecular profiles from 572 tumor samples and 8 normal controls. The molecular profile includes gene expression (microarray), genotype (SNP), exon expression, MicroRNA expression (microarray), copy number variation (CNV), DNA methylation, somatic mutation, gene expression (RNA-seq), MicroRNA-seq and protein expression. The clinical information includes records on recurrence, survival, and treatment resistance. These massive complex data sets have driven enthusiasm to study the molecular mechanism of cancers through computational approaches [1,6-8]. Among the developed methods, Bayesian Network (BN) is one of the most frequently used multivariate models. The BN approach is more appealing than graphs constructed based on correlation or mutual information metrics for it allows rigorous statistical inference of causality between genetic and epigenetic features. However most of the existing studies have been focused on one type of data either continuous or discrete [9-13]. How to combine different types of complex data for causal inference in BN poses a big challenge. In addition, deducing the complex network structure from data remains an open problem partially due to the lack of prior information, relatively smaller sample size and the high dimensionality of data (number of possible nodes) [13,14].

A necessary and important step to construct a BN from tens of thousands of features is feature selection, i.e., to identify a subset of the most-relevant features. Removing irrelevant or redundant features helps improve computing efficiency and estimation accuracy in the causal network. Existing feature selection methods can be roughly classified into two categories: wrapper approach [15,16] and filter approach [17-19]. For large data sets, the filter approach using significance test for difference between the cancer and control samples is more commonly used due to its simplicity. As some features could be causal to other features while having no direct association with the cancer phenotypes, the independent test can filter out many related features (see a simulation study in the Methods section). One innovation of this paper is a novel stepwise correlation-based selector (SCBS) that mimics the hierarchy of the BN for feature selection. The selected features from the TCGA data are a mixture of continuous and categorical variables. To integrate them into the same BN, we discretize the continuous variables and use a logit link function for casual inference. The proposed approach is applied to the TCGA ovarian cancer data and leads to a series of interesting findings that shed light into the genetic/epigenetic mechanisms of ovarian cancer.

## Results

### Preprocessing of TCGA ovarian cancer data

**Figure 1:**
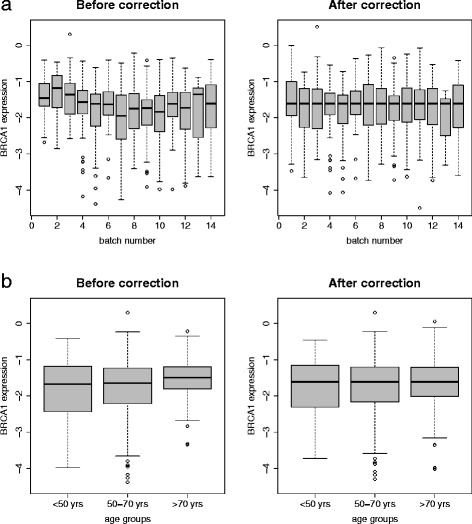
**Removal of batch effect and age effect.** **(a)** Boxplots of *BRCA1* expression before (on the left) and after (on the right) removal of batch effect, where x-axis is the batch number and y-axis is the expression level; **(b)** Boxplots of *BRCA1* expression before (on the left) and after (on the right) removal of age effect, x-axis is age group (<50 yrs old, 50-70 yrs old and >70 yrs old) and y-axis is the expression level. In the preprocessing step, we removed batch and age effects of expression level and methylation level for every single gene.

In this paper, we only consider four types of molecular data including gene expression, DNA copy number variation, promoter methylation and somatic mutation (summarized in Table 1). This data set contains the expression values of 17,812 genes, out of which, 12,831 had methylation level measured for each CpG island located in their promoter regions. If multiple CpG islands exist for a given gene, we took the average as the overall methylation level. The copy number was measured for each chromosomal segment, recorded as a seg.mean value, with the segment length varying from hundred up to tens of millions base pairs by the Circular Binary Segmentation (CBS) performed by TCGA. Out of 17,812 genes, 15,352 have well defined locations on the genome provided by UCSC Genome Browser (http://genome.ucsc.edu/) and each of them was assigned a value as the copy number. If a gene entirely falls within a chromosome segment, we assigned it the corresponding seg.mean value (236 out of 15,352 genes that spans two chromosomal segments were not assigned any seg.mean value). For the somatic mutation data, we defined a binary variable where "1" stands for all the non-silent mutations (coding different amino acid) and "0" for silent mutations or not being mutated, resulting in 9,895 genes with somatic mutations (mutation occurred in at least one sample). For the gene expression and methylation data, we applied an existing method by [8] to remove the effects due to different age groups and batches. Figure 1 illustrates an example for *BRCA1* gene where the boxplots showed this procedure was effective in removing the age and batch effects.

**Table 1:** **Summary of TCGA ovarian cancer data**

**Data type**	**Platform**	**Cases**
Gene expression	Agilent 244K	574 (8 organ-specific controls)
Somatic mutation	Agilent 415K	579 (8 organ-specific controls)
DNA methylation	Illumina 27K	584 (8 organ-specific controls)
Copy number variation	Agilent 1M	579 (8 organ-specific controls)
Clinical information	N/A	583

Summary of TCGA ovarian cancer data including the data types we incorporated in the analysis, platforms and the number of available cases.

The second step in data preprocessing is to discretize the continuous random variables. We classified the gene expression level into three groups using k-means clustering algorithm, namely *low*, *midium*, and *high*. Likewise the promoter methylation level was classified into either *hyper* or *hypo* state, and the copy number status into two states: *gain* or *loss*. The discretized variables were the input of the Bayesian network to be discussed below.

### Feature selection

**Figure 2:**
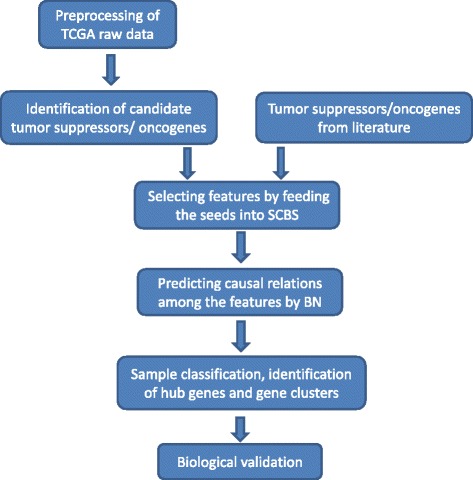
**Workflow of our integrative framework.**

The pipeline we propose in this paper assumes that cancer phenotype is directly associated with gene expression, which can be potentially driven by genetic and epigenetic changes (this assumption was made from the biological point of view. It can be dropped without affecting the modeling and computing). Figure 2 illustrates the workflow of our proposed framework. We first identify a set of tumor suppressors and oncogenes by differential expression analysis between the cancer and control groups. This set of genes, together with those that were previously reported in the literature as cancer-relevant, form the set of seed genes for further feature selection. These seed genes are then fed into our proposed stepwise correlation-based selector (SCBS) to select other features. The SCBS is motivated by the hierarchy of causality in the Bayesian network. For example, suppose there is a causal relationship A →B →cancer. Though A to B or B to cancer has strong directed association, the association between A and cancer could decay greatly so that it cannot be detected. The SCBS procedure starts with detection of features significantly correlated with the cancer and then progressively selects subsequent features that correlate with the selected features. Our simulation study presented in the Methods section shows that the proposed SCBS is more effective to select important features that are involved in the phenotype-related pathways but indirectly associated with the cancer phenotype.

The first step in feature selection is to define a set of seed genes out of 12,000 genes that have records of expression level and at least one of the three (epi)genetic factors (i.e., CNV, methylation and mutation). We conducted a nonparametric test to identify the most differentially expressed genes between case and control groups, as well as the most differentially methylated genes, most differentially mutated genes and genes with most differentiate copy numbers. A gene was defined as a candidate oncogene or tumor suppressor if it satisfies all the following three conditions: (1) the p-value of gene expression was statistically significant under Benjamini-Hochberg (BH) procedure with false discovery rate (FDR) ≤ 0.05; (2) the p-value of somatic mutation/promoter methylation/copy number was also significant under BH procedure with FDR ≤0.05; and (3) the absolute value of correlation coefficient between gene expression and somatic mutation/promoter methylation/copy number was greater than 0.4. The p-values were calculated using Wilcoxon rank-sum test and the correlation coefficients were calculated using Pearson’s method. This procedure resulted in 48 potential tumor suppressors or oncogenes (Table 2), 16 of which were well-studied tumor suppressors and oncogenes for ovarian cancer from literature (out of a total of 36 presented in [2], Table 3). The union of the two sets of 68 genes were defined as seed genes and generated an additional 271 nodes out of more than 50,000 candidate features by the stepwise correlation based selection (SCBS) procedure, which include 177 gene expressions, 82 copy number variation sites, 11 methylation sites and one somatic mutation site at gene *TP53*.

**Table 2:** **48 tumor suppressors and oncogenes from TCGA data**

**Oncogene/suppressor**	**Gene symbol**	**Name**
Suppressor	CDKN2A	Cyclin-Dependent Kinase Inhibitor 2A
Suppressor	MAP2K4	Mitogen-Activated Protein Kinase Kinase 4
Suppressor	MAGEC1	Melanoma Antigen Family C, 1
Suppressor	RIMBP2	RIMS Binding Protein 2
Suppressor	DIRAS3	DIRAS Family, GTP-Binding RAS-Like 3
Suppressor	PEG3	Paternally Expressed 3
Suppressor	DAB2	Disabled Homolog 2, Mitogen-Responsive Phosphoprotein
Suppressor	NF1	Neurofibromin 1
Suppressor	ARID1A	AT Rich Interactive Domain 1A
Suppressor	OPCML	Opioid Binding Protein/Cell Adhesion
Suppressor	PLAGL1	Pleiomorphic Adenoma Gene-Like 1
Suppressor	CASP9	Caspase 9, Apoptosis-Related Cysteine Peptidase
Suppressor	WWOX	WW Domain Containing Oxidoreductase
Suppressor	RPS6KA2	Ribosomal Protein S6 Kinase, 90kDa, Polypeptide 2
Suppressor	SPARC	Secreted Protein, Acidic, Cysteine-Rich
Suppressor	DLEC1	Deleted In Lung And Esophageal Cancer 1
Oncogene	THY1	Thy-1 Cell Surface Antigen
Oncogene	ALG3	Alpha-1, 3-Mannosyltransferase
Oncogene	ATP5E	ATP Synthase, H+ Transporting, Mitochondrial F1 Complex, Epsilon Subunit
Oncogene	ATP6V1C1	ATPase, H+ Transporting, Lysosomal 42kDa, V1 Subunit C1
Oncogene	C19orf53	Chromosome 19 Open Reading Frame 53
Oncogene	CSNK2A1	Casein Kinase 2, Alpha 1 Polypeptide
Oncogene	CTSF1	Cathepsin F
Oncogene	DERL1	Derlin 1
Oncogene	HSF1	Heat Shock Transcription Factor 1
Oncogene	ITPA	Inosine Triphosphatase
Oncogene	MRPL34	Mitochondrial Ribosomal Protein L34
Oncogene	NCBP2	Nuclear Cap Binding Protein Subunit 2
Oncogene	NDUFA13	NADH Dehydrogenase (Ubiquinone) 1 Alpha Subcomplex, 13
Oncogene	NDUFB7	NADH Dehydrogenase (Ubiquinone) 1 Beta Subcomplex, 7
Oncogene	NDUFB9	NADH Dehydrogenase (Ubiquinone) 1 Beta Subcomplex, 9
Oncogene	OSBPL2	Oxysterol Binding Protein-Like 2
Oncogene	POLR2H	Polymerase (RNA) II (DNA Directed) Polypeptide H
Oncogene	PIK3R1	Phosphoinositide-3-Kinase, Regulatory Subunit 1
Oncogene	AKT2	V-Akt Murine Thymoma Viral Oncogene Homolog 2
Oncogene	ERG	V-Ets Erythroblastosis Virus E26 Oncogene Homolog
Oncogene	PTK2	Protein Tyrosine Kinase 2
Oncogene	RAE1	RAE1 RNA Export 1 Homolog
Oncogene	RIOK1	RIO Kinase 1
Oncogene	SNRPB2	Small Nuclear Ribonucleoprotein Polypeptide B
Oncogene	SNX5	Sorting Nexin 5
Oncogene	SRXN1	Sulfiredoxin 1
Oncogene	STX10	Syntaxin 10
Oncogene	TRMT1	TRNA Methyltransferase 1 Homolog
Oncogene	TRMT6	TRNA Methyltransferase 6 Homolog
Oncogene	WDR53	WD Repeat Domain 53
Oncogene	YWHAZ	Tyrosine 3-Monooxygenase/Tryptophan 5-Monooxygenase Activation Protein, Zeta Polypeptide
Oncogene	RAB25	RAB25, Member RAS Oncogene Family

Presented in the table are the symbol and name of 48 tumor suppressors and oncogenes identified from TCGA data.

**Table 3:** **36 tumor suppressors and oncogenes from literature**

**Oncogene/suppressor**	**Gene symbol**	**Name**
Suppressor	RB1	Retinoblastoma 1
Suppressor	PTEN	Phosphatase And Tensin Homolog
Suppressor	DAB2	Disabled Homolog 2, Mitogen-Responsive Phosphoprotein
Suppressor	DLEC1	Deleted In Lung And Esophageal Cancer 1
Suppressor	TP53	Tumor Protein P53
Suppressor	NF1	Neurofibromin 1
Suppressor	SPARC	Secreted Protein, Acidic, Cysteine-Rich
Suppressor	TMPRSS2	Transmembrane Protease, Serine 2
Suppressor	CASP9	Caspase 9, Apoptosis-Related Cysteine Peptidase
Suppressor	PLAGL1	Pleiomorphic Adenoma Gene-Like 1
Suppressor	WWOX	WW Domain Containing Oxidoreductase
Suppressor	RPS6KA2	Ribosomal Protein S6 Kinase, 90kDa, Polypeptide 2
Suppressor	BRCA1	Breast Cancer 1, Early Onset
Suppressor	BRCA2	Breast Cancer 2, Early Onset
Suppressor	DIRAS3	DIRAS Family, GTP-Binding RAS-Like 3
Suppressor	PEG3	Paternally Expressed 3
Suppressor	ARID1A	AT Rich Interactive Domain 1A
Suppressor	OPCML	Opioid Binding Protein/Cell Adhesion
Oncogene	MYC	V-Myc Myelocytomatosis Viral Oncogene Homolog
Oncogene	CDC25A	Cell Division Cycle 25A
Oncogene	PIK3CA	Phosphatidylinositol-4, 5-Bisphosphate 3-Kinase
Oncogene	NOTCH3	Notch 3
Oncogene	EIF5A2	Eukaryotic Translation Initiation Factor 5A2
Oncogene	STAT3	Signal Transducer And Activator Of Transcription 3
Oncogene	ETV6	Ets Variant 6
Oncogene	EGFR	Epidermal Growth Factor Receptor
Oncogene	FGF1	Fibroblast Factor 1
Oncogene	AKT2	V-Akt Murine Thymoma Viral Oncogene Homolog 2
Oncogene	KRAS	V-Ki-Ras2 Kirsten Rat Sarcoma Viral Oncogene Homolog
Oncogene	RAB25	RAB25, Member RAS Oncogene Family
Oncogene	AURKA	Aurora Kinase A
Oncogene	PIK3R1	Phosphoinositide-3-Kinase, Regulatory Subunit 1
Oncogene	ERG	V-Ets Erythroblastosis Virus E26 Oncogene Homolog
Oncogene	ATAD2	ATPase Family, AAA Domain Containing 2
Oncogene	PDGFRA	Platelet-Derived Growth Factor Receptor, Alpha Polypeptide
Oncogene	ERBB2	V-Erb-B2 Erythroblastic Leukemia Viral Oncogene Homolog 2

Presented in the table are the symbol and name of 36 tumor suppressors and oncogenes reported in the literature [2].

### Bayesian network prediction

**Figure 3:**
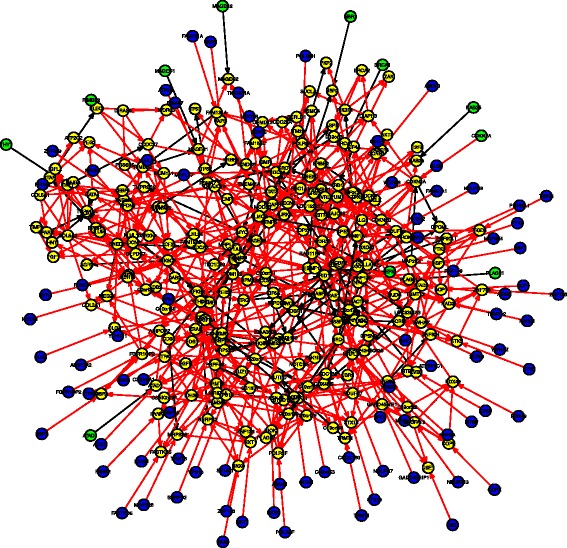
**Predicted graph by Bayesian network model with logit link function and blockwise coordinate descent algorithm, with 339 nodes including expression level of 245 genes (yellow), copy number at 82 sites (blue), methylation at 11 sites (green) and 1 somatic mutation at gene** ***TP53*** **, connected by 698 directed edges.** Direction of the edge indicates the downstream feature is regulated by the upstream one. Red edge represents activation and black edge represents suppression. Details are listed in Additional file 3: Table S3.

The 339 nodes (discretized if continuous) were fit into the Bayesian network through a logit link function using the blockwise coordinate descent algorithm for penalized maximum likelihood estimation procedure [20] (Methods).The predicted network contains 698 edges (Figure 3, details are tabulated in Additional file 1: Table S1), where the direction of the edge indicates the downstream feature is regulated by the upstream one. We found the CNVs are the major factor that accounts for differential gene expression. In addition most of the 82 genes were CNV-amplified in cancer samples, suggesting that many amplified genes may act as cancer drivers, confirming findings from a breast cancer study [21]. This network also confirmed many previously reported gene-gene interactions. To name a few, for example, the edge from *TPX2* to *AURKA* could be explained by the fact that the protein encoded by the *TPX2* gene activates *AURKA* by inducing autophosphorylation [22]. The connection between *BRCA1* and *NBR2* could be due to the shared bi-directional promoter between the two genes [23]. The connection between *STAT3* and *ETV6* was suggested previously that *ETV6* is a negative regulator of *STAT3* activity [24]. The edge from *CDKN2A* to *CCNE1* is a known gene-gene regulation in the RB signaling pathway [1]. The edge from *MYC* to *IMPHD2* confirms that *MYC* depletion results in repression of *IMPHD2* (a gene coding rate-limiting enzyme) [25]. These results suggested that the proposed pipeline is capable of revealing important genetic or epigenetic pathways that underlie the complex cancer phenotype.

**Figure 4:**
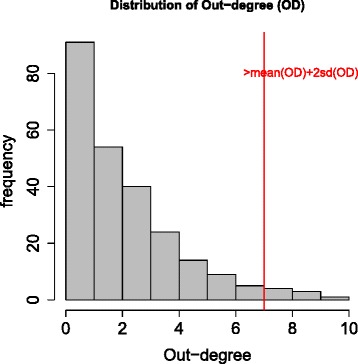
**Histogram of outdegree (number of edges going out from the node) for each gene in the predicted network, the mean degree and standard deviation are 2.15 and 2.31 respectively.** Genes with outdegree greater than 7 (mean+2SD) are identified as hub genes. The 13 hub genes are listed in Table 4.

**Figure 5:**
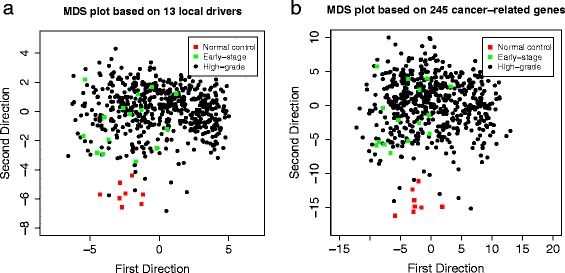
**Multidimensional Scaling (MDS) plots for sample classification.** **(a)** MDS plot based on 13 hub genes only where the distance between samples is measured by Euclidean distance of the gene expression level; **(b)** MDS plot based on all the 245 genes in the predicted network where each dot represents one sample and totally 580 samples including 8 normal samples (cancer-free, red), 15 early-stage samples (cancer at stage I, green) and 557 high-grade samples (cancer at stage II or higher, black).

The average degree (indegree plus outdegree) of the graph is 4.124 indicating that the inferred network is sparse with moderate complexity. Due to the directionality of BN, one could also obtain the indegree and outdegree separately for every single node. Figure 4 shows the observed distribution of outdegree, where the mean and standard deviation are 2.15 and 2.31 respectively. We identified 13 nodes with significantly larger outdegree (greater than mean + 2 × SD, ≥7 edges) in the network including *ARID1A*, *C19orf53*, *CSNK2A1*, *DERL1*, *TRMT6*, *COL5A2*, *TCF21*, *LUM*, *TPX2*, *UBE2C*, *DPM1*, *NDUFB7*, and *NDUFB9* (Table 4). These hub genes all have known functions and have causal effect on at least seven neighboring genes, suggesting that they may play important roles in driving corresponding local subnetworks. Some of the hub genes have been reported in the literature that are related to ovarian cancer. For instance, *ARID1A* is known to promote the formation of SWI/SNF chromatin remodeling complexes containing *BRG1* or *BRM*, and is a candidate tumor suppressor not only in clear cell ovarian cancer but also in endometrioid cancers and uterine endometrioid carcinomas [22,26,27]. *C19orf53* is known to be associated with Leydig cell tumors which are a member of the sex cord-stromal tumor group of ovarian and testicular cancers and it has a potential role in hypercalcemia of malignancy [22]. *CSKN2A1* is a well-known oncogene that can phosphorylate a number of key intracellular signaling proteins implicated in tumor suppression (*P53* and *PTEN*) and oncogenesis (*MYC*, *JUN*, *NF-kappaB*). This gene also influences Wnt signaling via beta-catenin phosphorylation and the *PI3K* signaling pathway via the phosphorylation of *AKT* [22]. Interestingly these 13 hub genes can clearly distinguish the cancer samples from the normal samples as revealed by a multi-dimensional scaling plot (MDS, [28]) based on the correlation dissimilarity metric (comparable clustering effect was observed based on the entire set of 245 genes, Figure 5a,b). This suggests that the thirteen hub genes may present the major difference between the cancer and normal samples. The early-stage and high-grade tumor samples however are not well distinguished.

**Table 4:** **13 local drivers (hub genes) in the predicted network**

**Gene symbol**	**Name**
ARID1A	AT Rich Interactive Domain 1A
C19orf53	Chromosome 19 Open Reading Frame 53
CSNK2A1	Casein Kinase 2, Alpha 1 Polypeptide
DERL1	Derlin 1
TRMT6	TRNA Methyltransferase 6 Homolog
COL5A2	Collagen, Type V, Alpha 2
TCF21	Transcription Factor 21
LUM	Lumican
TPX2	Microtubule-Associated, Homolog
UBE2C	Ubiquitin-Conjugating Enzyme E2C
DPM1	Dolichyl-Phosphate Mannosyltransferase Polypeptide 1,
	Catalytic Subunit
NDUFB7	NADH Dehydrogenase (Ubiquinone) 1 Beta Subcomplex, 7
NDUFB9	NADH Dehydrogenase (Ubiquinone) 1 Beta Subcomplex, 9

Presented in the table are the symbol and name of 13 hub genes identified from the predicted Bayesian network.

### Gene clusters

**Figure 6:**
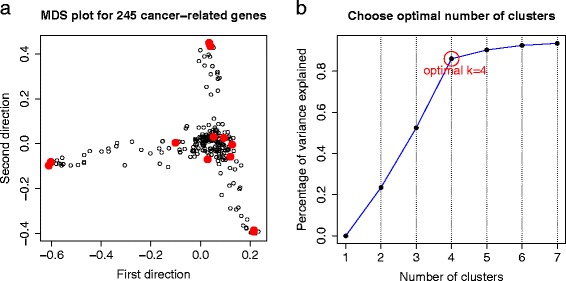
**Identification of gene clusters.** **(a)** MDS plot based on correlation dissimilarity metric among 245 genes (each circle represents one gene), where 13 hub genes are indicated by red dots; **(b)** The proportion of variance that can be explained by clustering (y-axis) against the number of clusters (x-axis) based on different values of k (k =1,2,…,7) by k-means clustering method. From this plot, the most likely number of clusters is four.

**Figure 7:**
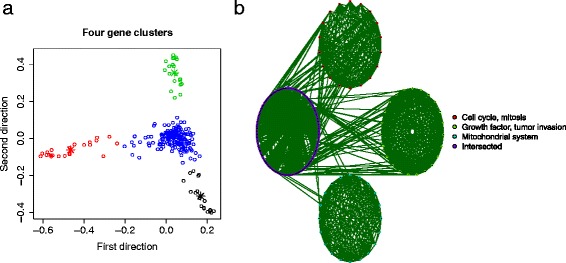
**Four major clusters with distinct cellular functions.** **(a)** Multidimensional (MDS) plot based on correlation dissimilarity metric between 245 genes (each circle represents one gene). Genes falling into four clusters (by k-means clustering method where k = 4) are indicated by different colors; **(b)** Correlation plot of the four clusters, the connection between a pair of genes represents a significant correlation.

The 245 genes (listed in Additional file 2: Table S2) were identified to fall into four major clusters corresponding to distinct functions by k-means clustering method (Figure 6b). Cluster 1 (black in Figure 7a) contains 18 genes, mainly related to cell division, mitosis, spindle formation etc. Cluster 2 (red) contains 23 genes, most of which are functionally related to growth factor, cell shape, cell motility, tumor invasion etc. Cluster 3 (green) contains 20 genes, mostly related to mitochondrial system, membrane process etc. Cluster 4 (blue in Figure 7a) is the largest and most complicated cluster harboring 184 genes. This large cluster communicates between the other three clusters (as shown in Figure 7b), which are nearly independent from each other. This can be seen from the summary statistics of within and between cluster causal edges in Table 5. These findings could be implicative of some important molecular pathways, which may or may not have been identified, that drive the development of ovarian cancer.

**Table 5:** **Number of causal edges within/between four clusters in the TCGA ovarian cancer data**

	**Cluster 1**	**Cluster 2**	**Cluster 3**	**Cluster 4**
	**(23)**	**(18)**	**(20)**	**(184)**
Cluster 1 (23)	46	0	2	35
Cluster 2 (18)		28	0	40
Cluster 3 (20)			40	29
Cluster 4 (184)				384

Presented in the table are the number of predicted edges within and between clusters. The number of genes in each cluster is listed in the parentheses.

**Figure 8:**
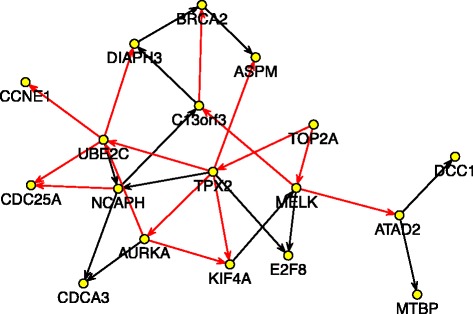
**Subnetwork extracted from Figure** 3 **which is corresponding to cell division process (mitosis, spindle formation etc), containing 18 nodes and 28 directed edges.** Direction of the edge indicates the downstream feature is regulated by the upstream one. Red edge represents activation and black edge represents suppression. *TPX2* and *UBE2C* are two hub genes that may drive this subnetwork.

We also looked into the subnetwork within each cluster. Figure 8 shows the local subnetwork corresponding to the first cluster which is involved mainly in cell division processes. Two hub genes, *TPX2* and *UBE2C*, are in the central positions of this network. Our finding that there are four gene clusters may suggest that the development of ovarian cancer could be partially driven by the cell cycle regulation, as well as the pathways related to cell shape and motility, and mitochondrial system. Intervention (activation or suppression) on the hub genes or other important genes may alter the entire network, therefore may control key aspects of disease development.

### Survival-centric network prediction

**Figure 9:**
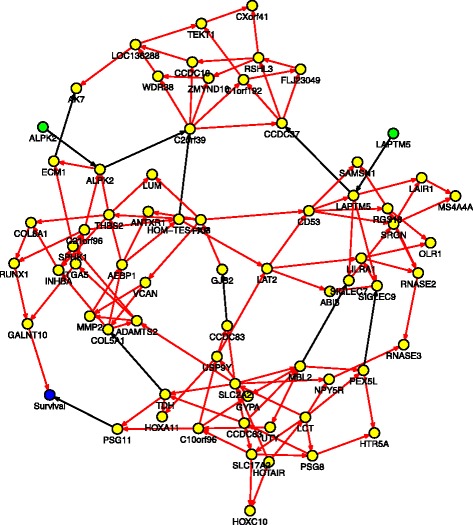
**Survival induced Bayesian network, including expression level of 66 genes (yellow) and promoter methylation at 2 sites (green).** Two genes, *PSG11* and *GALNT10*, have direct effect on the survival time. Direction of the edge indicates the downstream feature is regulated by the upstream one. Red edge represents activation and black edge represents suppression. Details are listed in Additional file 3: Table S3.

**Figure 10:**
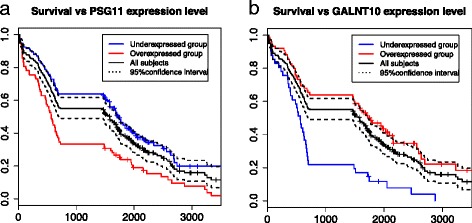
**Survival probability against time of different groups by the expression level of gene** ***GALNT10*** **(a) and gene** ***PSG11*** **(b).** The x axis represents the survival time (in days) and y axis represents survival rate. By log-rank test, the p-values are 4.9×10^−5^ and 2.2×10^−9^ for *PSG11* and *GALNT11*, respectively. The black solid line is based on all subjects (235 patients) and the 95% confidence limits are represented by the black dashed lines. The red and blue lines are based on overexpressed and underexpressed group, respectively.

We applied SCBS and Bayesian network model to construct a survival-centric network. In feature selection, we used continuous data to select genetic/epigenetic features that were most relevant to the overall survival time of ovarian cancer patients. Starting from the first node (overall survival time in days), the SCBS procedure selected 66 genes (Table 6) and two methylation sites as the nodes in the Bayesian Network. Interestingly only 6 genes including *CCDC19*, *MMP1*, *SLC* family, *TEKT2*, *WDR* family and *ZMYND10* had been reported relevant to cancer survival in a separate study (where a total of 88 genes were reported, [29]). The death risk (binary) is then used as the phenotype node to build a phenotype-induced network. We defined the overall survival time for less than 2 years as the "high-risk" (134 patients) and the survival time greater than 4 years as the "low-risk" (101 patients). In our predicted graph, there are 9 hub genes (similarly defined as above) that drive corresponding local subnetworks including *C2orf39*, *FAP*, *SLC2A2*, *LAPTM5*, *CD53*, *THBS2*, *CCDC63*, *SLC17A*, and *LCT*. Gene *FAP* has a known function to control fibroblast growth or epithelial-mesenchymal interactions during development, tissue repair, and epithelial carcinogenesis [22]. The inferred Bayesian network (Figure 9, details are tabulated in Additional file 3: Table S3) identified two genes, namely *PSG11* and *GALNT10*, that may be directly associated with the overall survival time of ovarian cancer patients (Figure 10). Both genes are functionally related to glycoprotein synthesis, as well as many other genes in the network such as *SLC2A2*, *SLC17A*, *CD53*, *THBS2*, *LCT*, *GYPA* [22]. This indicates the biological pathway related to glycoprotein synthesis may be implicative of death risk of ovarian cancer patients. As reported in literature [30,31], several tumor-associated glycoproteins were found on the surface of many cancer cells including ovary, breast, colon, and pancreatic cells and they may play potential roles in early detection of caners. One well-known such glycoprotein is *CA-125* (encoded by gene *MUC16*), which is the primary protein used to measure serous cancer tumor load, especially during recurrence, and it is heavily glycosylated [31]. Pregnancy-specific glycoproteins (PSG) are mainly produced by the placental syncytiotrophoblasts during pregnancy and these proteins comprise a subgroup of the carcinoembryonic antigen family [22]. The protein encoded by gene *GALNT10* may have increased catalytic activity toward glycosylated peptides compared to activity toward non-glycosylated peptides [22]. As pointed out by several research groups [32-34], some certain glycoproteins are closely associated with women cancers such as ovarian cancer and breast cancer, affecting the death risk, chemotherapy resistance and prognosis of ovarian cancer patients. The network also involves genes of other important functions including microtubules (*RSHL3*, *TEKT1* and *OLR1*), extracelluar processes (*ECM1*, *THBS2*), hematopoietic (*MS4A4*, *SRGN*, *LAPTM5*), and human immune system (*LILRA1*, *SIGLEC7*, *LAT2*, *LAIR1*). This network suggested many causal relationship between different features, some of which have been known, for example, the edge *HOTAIR* →*HOXC10* may be due to the fact that *HOTAIR* (a noncoding RNA gene) is located within the Homeobox C (*HOXC*) gene cluster and it regulates the expression of HOX genes such as *HOXC* and *HOXD* [22,35].

**Table 6:** **66 survival-related genes**

AK7	C2orf39	CCDC19	LOC136288	WDR38	C1orf192	CCDC37
FLJ23049	RSHL3	TEKT1	CXorf41	ZMYND10	RNASE3	LILRA1
MS4A4A	RNASE2	SIGLEC7	ABI3	LAT2	OLR1	SIGLEC9
CD53	LAIR1	SAMSN1	SRGN	RGS18	LAPTM5	PSG11
C10orf96	HOTAIR	HTR5A	TDH	CCDC83	GYPA	USP9Y
UTY	HOXC10	LCT	NPY5R	SLC2A2	MBL2	PEX5L
PSG8	SLC17A2	CCDC63	HOXA11	GALNT10	GJB2	ITGA5
MMP2	RUNX1	FAP	INHBA	THBS2	VCAN	ADAMTS2
ALPK2	ECM1	SPHK1	AEBP1	COL5A1	LUM	ANTXR1
C21orf96	COL8A1	HOM-TES-103

## Discussion

In this paper we proposed an integrative approach in the Bayesian network framework for causal inference between genetic and epigenetic features in complex cancer data. It presents novelty in two aspects. First, we demonstrated that the stepwise correlation-based selection approach is more effective than simple single-round selection method in identifying important features in the genetic/epigenetic pathways, particularly those that are indirectly associated with the underlying phenotype. The method we proposed relies on the correlation strength among connected nodes and may fail when the connections are weak, especially for high-dimension data where assigning significance is challenging. Using literature-verified genes as seeds helps to better select features relevant to the phenotype. The SCBS procedure is model-free and computationally efficient and it can be applied to other graphical models such as Markov Random Field (MRF, undirected graph) and gene-gene or protein-protein interaction (PPI) network problems.

Second, we proposed a method for modeling causal relationships between features of different types (continuous or discrete) in a Bayesian network through a logit link function. The block-wise coordinate descent (BCD) algorithm accompanied with the Bayesian network model provides a simple and efficient way to estimate the parameters in the model. With a moderate sample size, this method achieves reasonable accuracy even for a moderate-scale network containing 200 nodes. This pipeline can be readily applied to other complex cancer data for pathway analysis or to find the common pathways between different but correlated phenotypes. For example, the TCGA now has accumulated more than 1,000 samples from breast cancer patients. It would be highly intriguing to know whether these two common diseases share any common molecular basis, especially for the basal-like breast cancer. As previously reported in literature [36], the basal-like subtype of breast cancer is the most distinct of the four subtypes (Luminal A, Luminal B, HER2-enriched and Basal-like) and it is similar to high-grade serous ovarian cancer at the mRNA expression level.

The Bayesian network model allows strict probabilistic inference, but also has limitations. First, it lacks flexibility to model cyclic causal relationships. The BN cannot model any cyclic pathways, for example, A →*B*→*C*→A, which though may exist in gene regulation *invivo*. Second, most of the existing BN learning algorithms assume sparsity for computational feasibility. If the true network is dense or locally dense (dense subnetworks), the weak causations may fail to be detected. Our proposed BN with logit link function can model categorical variables and discretized continuous variables simultaneously, while discretization procedure may cause loss of information. A more desirable way could be to model the discrete variable nodes with logit distribution, but the continuous variable nodes with Gaussian or other continuous distributions. This direction demands future research. The coordinate descent is successful in solving the LASSO-type problem, especially in the sparse BN problem. Due to the super-exponentially increased number of possible BNs, the traditional sampling-based methods such as Metropolis-Hastings algorithm ([13,14]) is computational infeasible to estimate the network with moderate number of nodes, e.g., 100 nodes. In our problem involving 339 nodes, the BCD algorithm took about 20 minutes on a single CPU to complete the estimation for a given penalizing parameter *λ* (Materials and Methods).

We illustrated the flexibility of this pipeline with two phenotypes, namely the ovarian cancer phenotype itself and the survival time of the cancer patient. The induced network by the cancer phenotype contains a set of 245 genes forming into four major clusters of distinct major functions, coordinated by 13 hub genes. Some of the hub genes (e.g., *ARID1A*) have been reported by other researchers for their important roles in genetic pathways, while other genes (*NDUFB7* and *NDUFB9*) are newly discovered in this study. Their functions in ovarian cancer need to be further investigated. Our discovery that the mitochondrial systems are regulated in serous tumors is consistent with the hypothesis that the Warburg effect impacts tumor progression as suggested in published studies ([37]). We also found that pathways related to glycoprotein synthesis, hematopoietic and immune systems correlate with the survival rate of ovarian cancer patients. In particular, we discovered that the two genes related to glycoprotein synthesis, *PSG11* and *GALNT10*, can significantly affect the overall survival time of ovarian cancer patients.

## Conclusions

Understanding the biological mechanism of ovarian cancer has significant practical importance for clinical diagnosis and treatment. Timely advent of TCGA project has provided the most comprehensive genomic data resource for cancer study at the molecular and system level. Nevertheless, how to utilize these complex data for discovery of molecular mechanism of cancers remains one of the biggest challenges in this field. To this end, we propose a new integrative approach in this paper, which presents two innovations: a stepwise feature selection procedure and a Bayesian network model that incorporates both continuous and discrete features for causal inference. The predicted graph for the ovarian cancer data confirmed numerous genetic pathways reported in the literature, as well as many new ones that may provide new clues to guide future research. The graph suggested 13 hub genes that may drive certain subnetworks therefore play important roles in ovarian cancer. Clustering analysis suggested four gene clusters corresponding to distinct biological processes including cell division, tumor invasion and mitochondrial system. In addition, we found that genes related to glycoprotein synthesis, hematopoietic, immune system could be highly predictive of overall survival time of ovarian cancer patients.

## Methods

### Data

Datasets in ovarian cancer were downloaded from the Cancer Genome Atlas (TCGA) data portal (http://tcga-data.nci.nih.gov). We extracted four types of molecular data including gene expression, promoter methylation, somatic mutation and DNA copy number variation, using the "data matrix" tool provided by TCGA data portal.

### Bayesian network with a logit link function

Bayesian Network can be used to model a set of random variables (nodes) and their conditional dependencies (directed edges) [11]. In general, the joint likelihood function of nodes *X*_1_,…,*X*_*p*_ in a BN can be expressed as:

(1) $$ P(X_{1},\ldots,X_{p})=\prod\limits_{i=1}^{p}P\left(X_{i}|\Pi_{i}^{\mathcal{G}}\right)  $$

where graph $\mathcal {G}=(V,E)$ represents the topological structure of the Bayesian network, *V*={*X*_1_,…,*X*_*p*_} denotes the set of nodes and $E=\left \{X_{j} \rightarrow X_{i}, X_{j}\in \prod _{i}^{\mathcal {G}}\right \}$ denotes the set of edges, and $\Pi _{i}^{\mathcal {G}} \subseteq \left \{X_{1},\ldots,X_{p}\right \}\setminus \left \{X_{i}\right \}$ stands for the parent set of *X*_*i*_. We say $X_{j} \in \Pi _{i}^{\mathcal {G}}$ if *X*_*j*_ causes *X*_*i*_, written as *X*_*j*_→*X*_*i*_. A BN is called Gaussian Bayesian Network (GBN) if *X*_*i*_ is normally distributed with the mean equal to a linear combination of $X_{j}\in \Pi _{i}^{\mathcal {G}}$. The GBN is the most popular BN model and its structure learning problem has been discussed by several researchers [20,38]. In our motivating example, however the network involves both continuous and discrete random variables. Here we discretize the continuous random variable and consider a multinomial logistic model.

Let *X*_*i*_ take values from {1,…,*K*_*i*_} with probabilities $\left \{\pi _{i1},\ldots,\pi _{{iK}_{i}}\right \} \left (\mathrm {s.t.} \sum \limits _{k=1}^{K_{i}}\pi _{\textit {ik}}=1\right)$, the BN model with logit link function can be written as:

(2) $$ \log\frac{\pi_{ik}}{\pi_{{iK}_{i}}}=\upbeta_{ik0}+\sum\limits_{j\ne i}\sum_{l=1}^{K_{j}-1}\upbeta_{ikjl}\mathbf{I}\left\{X_{j}=l\right\},  $$

where *k*=1,…,*K*_*i*_−1 and *i*=1,…,*p*. Here we transform the network structure to a coefficient matrix where *β*_*ikjl*_=0 for all *k* and *l* means *X*_*j*_↛*X*_*i*_, and otherwise *X*_*j*_→*X*_*i*_. Therefore estimating the structure of  is equivalent to estimating matrix {*β*_*ikjl*_}. For simplicity, we illustrate the parameter estimation using binomial logistic model where all *X*_*i*_’s only take values 0 or 1. Define *π*_*i*_≡ prob (*X*_*i*_=1), then

(3) $$ \log\frac{\pi_{i}}{1-\pi_{i}}=\upbeta_{i0}+\sum\limits_{j\ne i}\upbeta_{ij}X_{j}.  $$

Suppose we observe data from *N* subjects. Let **X**_*n*_=(*X*_*n*0_,*X*_*n*1_,…,*X*_*np*_)^*T*^, where *X*_*n*0_=1 is the dummy variable, and *X*_*ni*_=0 or 1 for *n*=1,…,*N*;*i*=1,…,*p*. Define *π*_*ni*_≡ prob (*X*_*ni*_=1). Let $\mathbf {X}_{n_{-i}}=(X_{n0},\ldots,X_{n(i-1)}, X_{n(i+1)}\ldots,X_{\textit {np}})^{T}$, and ***β***_*i*_=(*β*_*i*0_,…,*β*_*i*(*i*−1)_,*β*_*i*(*i*+1)_,…,*β*_*ip*_)^*T*^, then:

(4) $$ \pi_{ni}=\exp\left({\upbeta_{i}^{T}}\mathbf{X}_{n_{-i}}\right)\left/\left(1+\exp\left({\upbeta_{i}^{T}}\mathbf{X}_{n_{-i}}\right)\right)\right..  $$

To achieve the sparsity, we apply the *L*_1_ penalty to the log-likelihood ([20,38]) as follows:

(5) $$  \begin{aligned} L^{*}(\upbeta)&=\sum\limits_{n=1}^{N}\sum\limits_{i=1}^{p}\left(X_{ni}{\upbeta_{i}^{T}}\mathbf{X}_{n_{-i}}-\log \left(1+\exp\left({\upbeta_{i}^{T}}\mathbf{X}_{n_{-i}}\right)\right)\right)\\ &\quad-\lambda\sum\limits_{i=1}^{p}\|\upbeta_{i}\|_{L_{1}}. \end{aligned}  $$

We aim to optimize the objective function (5) under the constraint of acyclicity. Finding the global maximizer is typically difficult in such a high dimensional space. Here we consider the coordinate descent (CD) algorithm, which has been successfully used to solve lasso regression problems [20,39]. The CD algorithm is based on single-parameter updating strategy to minimize the objective function coordinate-by-coordinate. For our model, the single-parameter updating can be done as follows, in particular, we seek the maximizer $\hat {\upbeta }_{\textit {ij}}$ of the following objective function given all the other parameters, denoted *β*_−*ij*_:

(6) $$  \begin{aligned} L^{*}_{i}\left(\upbeta_{ij}|\upbeta_{-ij}\right)&=\sum\limits_{n=1}^{N}\left(X_{ni}{\upbeta_{i}^{T}}\mathbf{X}_{n_{-i}}\,-\, \log\!\left(1+\exp\left({\upbeta_{i}^{T}}\mathbf{X}_{n_{-i}}\right)\!\right)\!\right)\\ &\quad-\lambda|\upbeta_{ij}|. \end{aligned}  $$

After excluding the constant part, we have:

$$ L^{*}_{i}\left(\upbeta_{ij}|\upbeta_{-ij}\right)\,=\,C_{1}\upbeta_{ij}-\lambda|\upbeta_{ij}|-\sum\limits_{n=1}^{N}\log\left(1+C_{2n} \exp\left(C_{3n}\upbeta_{ij}\right)\!\right)\!. $$

where $C_{1}=\sum \limits _{n=1}^{N}X_{\textit {ni}}X_{\textit {nj}}\geq 0$, $C_{2n}=\exp \left (\sum \limits _{k\neq i, j}\upbeta _{\textit {ik}}X_{\textit {nk}}\right)> 0$, *C*_3*n*_=*X*_*nj*_≥0. Note that $L^{*}_{i}\left (\upbeta _{\textit {ij}}|\upbeta _{-ij}\right)$ is concave and differentiable at (−*∞*,0)∪(0,*∞*). Let $\mathrm {f}\left (\upbeta _{\textit {ij}}\right)=-\sum \limits _{n=1}^{N} \log \left (1+C_{2n}\exp \left (C_{3n}\upbeta _{\textit {ij}}\right)\right)$, do the following to find the maximizer of $L^{*}_{i}\left (\upbeta _{\textit {ij}}|\upbeta _{-ij}\right)$:

1. If $\mathrm {f}^{\prime }(\upbeta _{\textit {ij}})|_{\upbeta _{\textit {ij}}=0} \in (-\infty, -C_{1}-\lambda)$, then $L^{*}_{i}(\upbeta _{\textit {ij}}|\upbeta _{-ij})$ is decreasing at 0 and $\hat {\upbeta }_{\textit {ij}}<0$. Find $\hat {\upbeta }_{\textit {ij}}$ by Newton’s method;
2. If $\mathrm {f}^{\prime }(\upbeta _{\textit {ij}})|_{\upbeta _{\textit {ij}}=0} \in (-C_{1}-\lambda, -C_{1}+\lambda)$, then $L^{*}_{i}(\upbeta _{\textit {ij}}|\upbeta _{-ij})$ is increasing on (−*∞*,0) and decreasing on (0,*∞*), so $\hat {\upbeta }_{\textit {ij}}=0$;
3. If $\mathrm {f}^{\prime }(\upbeta _{\textit {ij}})|_{\upbeta _{\textit {ij}}=0} \in (-C_{1}+\lambda, \infty)$, then $L^{*}_{i}(\upbeta _{\textit {ij}}|\upbeta _{-ij})$ is increasing at 0 and $\hat {\upbeta }_{\textit {ij}}>0$. Find $\hat {\upbeta }_{\textit {ij}}$ by Newton’s method.

The acyclicity constraint brings a major difficulty in BN learning problem, especially when the topological order of nodes is unknown. One immediate result of this constraint is that *β*_*ij*_ and *β*_*ji*_ cannot be both nonzero. To take advantage of this implication, Fu and Zhou (2013) proposed a blockwise coordinate decent (BCD) algorithm where the *p*(*p*−1) parameters are partitioned in to *p*(*p*−1)/2 blocks. Each block consists of *β*_*ij*_ and *β*_*ji*_. The BCD algorithm [20] can be implemented as follows (starting from an empty network where all *β*_*ij*_=0):

- Step 1: For each pair of *β*_*ij*_ and *β*_*ji*_, $\hat {\upbeta }_{\textit {ij}} \Leftarrow 0$ stands for that $\hat {\upbeta }_{\textit {ij}}$ has to be 0 under the constraint of acyclicity:
  - If $\hat {\upbeta }_{\textit {ji}} \Leftarrow 0$, find the maximizer $\hat {\upbeta }_{\textit {ij}}$ of $L^{*}_{i}$ w.r.t. *β*_*ij*_.
  - If $\hat {\upbeta }_{\textit {ij}} \Leftarrow 0$, find the maximizer $\hat {\upbeta }_{\textit {ji}}$ of $L^{*}_{j}$ w.r.t. *β*_*ji*_.
  - If either $\hat {\upbeta }_{\textit {ij}}$ or $\hat {\upbeta }_{\textit {ji}}$ can be nonzero, then compare the two sums: $S_{1}=L^{*}_{i}|_{\upbeta _{\textit {ij}}=0}+L^{*}_{j}|_{\upbeta _{\textit {ji}}=\hat {\upbeta }_{\textit {ji}}}$ and $S_{2}=L^{*}_{i}|_{\upbeta _{\textit {ij}}=\hat {\upbeta }_{\textit {ij}}}+L^{*}_{j}|_{\upbeta _{\textit {ji}}=0}$. Find maximizer of max(*S*_1_,*S*_2_).
- Step 2: Repeat step 1 until the maximum difference between two successive cycles is below some threshold.

To check the acyclicity of the candidate graph after edge *i*→*j* is added, we use a simple breadth-first algorithm detailed as follows. The time complexity of this algorithm is *O*(|*V*|+|*E*|):

- Step 1: Remove all the edges coming into *j* and identify the children set of *j*, denoted by *CS*_*j*_.
- Step 2: If *i*∈*CS*_*j*_, then stop. Otherwise remove all the edges coming into *CS*_*j*_ and find the children set of *CS*_*j*_.
- Step 3: Repeat until *i* is found or all the edges were removed.

Notice that in multinomial setting, the acyclicity constraint forces multiple *β*’s to be zero. For instance, in ternary case, *X*_*i*_→*X*_*j*_ indicates that four *β*’s are zeroes simultaneously. Another important issue in Lasso regression is the choice of the tuning parameter *λ*. Cross-validation is the most commonly used method for selecting *λ*, which however, tends to select a too small *λ* resulting in high false positive rate ([20]). To overcome this difficulty, an empirical *λ* selection method was proposed in [20] that can guarantee significant increase of the maximized likelihood value as a function of the graph complexity (number of edges). We employed this method for the selection of *λ* and the sequence of candidates is set to be {1/16,1/8,1/4,1/2,1,2,4,8,16}.

### Simulation I: BCD algorithm for BN with discrete nodes

In the first simulation, we evaluated the performance of BCD algorithm in the proposed Bayesian network with logit link function as follows. We first simulated a random graph (i.e., $\mathcal {G}=(V,E)$) with *p* nodes and 2*p* edges respectively. The simulated graph is a weakly connected directed graph generated using R package **bnlearn** ([40]). Given each , we simulated *N* independent observations, i.e., **X**_*n*_=(*X*_*n*1_,…,*X*_*np*_), for *n*=1,..,*N*. If *X*_*nj*_ is caused by other nodes, then we simulated it based on Bernoulli distribution with success probability following the logistic regression model. For simplicity, for each observation the causal effect *β*_*ij*_ (if *X*_*i*_→*X*_*j*_ in ) in the logistic regression model was set as a constant. For those nodes in the network but not caused by any other nodes, we simulated them from binomial distribution independently with success probability randomly generated from uniform distribution from 0.1 to 0.9. In this simulation we consider a crossed design of *p*=50,100,200, *β*_*ij*_=0.5,1 and *N*=500,1000,2000. For each setting of (*p*,*β*_*ij*_,*N*), 10 replicated samples were generated. We evaluated the estimated network under two different criteria: the directed version and undirected version of network (skeleton). In the former, we count an edge as a true positive only if it has the correct link and direction. For the skeleton comparison, an edge is counted as a true positive as long as it has the correct link. Table 7 presents the average number of predicted edges (P) from the 10 replicates for each setting, true positive rate (TPR) and false discovery rate (FDR) for both directed and undirected edges. Unsurprisingly the estimation of network structure is affected by the complexity of network, magnitude of causal effect and sample size. For a sparse network with 200 nodes, and sample size *N*=2000, and *β*=1, the BCD algorithm achieves an average TPR of 0.7 and 0.96 for directed and undirected (skeleton) graphs respectively. This simulation demonstrates that the BCD algorithm performs reasonably well when applied to categorical data in a moderate complex network when the sample size is relatively large.

**Table 7:** **Simulation I results**

***p***	***|E|***	***β***	**N**	**P**	**TPR (skeleton)**	**FDR (skeleton)**
50	100	0.5	500	46.0	0.298 (0.410)	0.468 (0.152)
			1000	63.2	0.420 (0.627)	0.333 (0.063)
			2000	78.4	0.600 (0.783)	0.273 (0.032)
		1	500	78.2	0.550 (0.740)	0.294 (0.051)
			1000	92.8	0.676 (0.910)	0.265 (0.019)
			2000	98.4	0.781 (0.960)	0.236 (0.017)
100	200	0.5	500	110.6	0.260 (0.400)	0.528 (0.272)
			1000	124.2	0.328 (0.557)	0.484 (0.104)
			2000	168.4	0.590 (0.825)	0.291 (0.019)
		1	500	163.2	0.539 (0.735)	0.349 (0.098)
			1000	167.8	0.614 (0.892)	0.347 (0.018)
			2000	194.4	0.768 (0.959)	0.216 (0.010)
200	400	0.5	500	252.6	0.225 (0.358)	0.647 (0.444)
			1000	272.8	0.383 (0.597)	0.438 (0.132)
			2000	326.4	0.546 (0.791)	0.337 (0.031)
		1	500	347.2	0.535 (0.825)	0.377 (0.073)
			1000	364.8	0.583 (0.872)	0.359 (0.044)
			2000	396.4	0.698 (0.963)	0.294 (0.028)

Presented in the table are the average number of predicted edges (P), true positive rate (TPR) and false discovery rate (FDR) for both directed and undirected (skeleton) edges over 10 replicated samples in each setting (*p* |*E*|, *β*, *N*). The number of edges |*E*| is set to be 2*p*.

### Comparison of three BN models on real data

**Figure 11:**
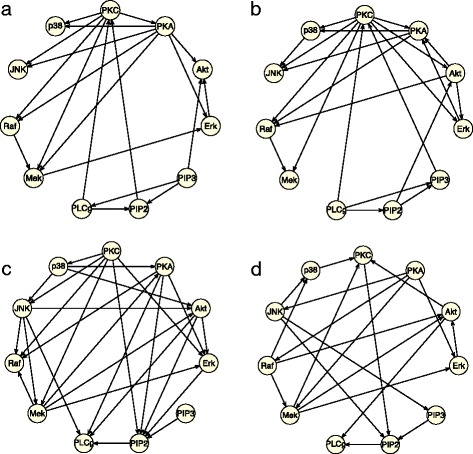
**Comparison of three different Bayesian network models.** **(a)** The known signaling pathway (Bayesian network) containing 11 proteins (nodes) and 20 causal relations (directed edges); **(b)** Predicted network by logistic BN model; **(c)** Predicted Network by Gaussian BN model; **(d)** Predicted network by Multinomial BN model.

To benchmark our logistic BN model, we compared it with two other BN models, namely the Gaussian BN model and multinomial BN model, on a popular data set [41] where the true causal network is known and experimentally-validated. This data set contains the abundance measurement of 11 proteins in 5400 samples, and has been used to elucidate the signaling pathway structure. Both the continuous and discrete versions of data are available online. The known protein-protein network is a Bayesian Network containing 11 nodes and 20 directed edges. Because this data set is based on experimental interventions, we adapt our model by deleting the intervention terms from the likelihood function. Figure 11 shows the true graph and estimated graphs by three different models and Table 8 summarizes the true positive rate and false discovery rate by three models. In terms of TPR and FDR, the logistic model appears to perform slightly better than other two models.

**Table 8:** **Comparison of three different BN models**

**Model**	**P**	**TPR (skeleton)**	**FDR (skeleton)**
Gaussian BN	26	0.55 (0.70)	0.58 (0.46)
Multinomial BN	20	0.40 (0.60)	0.60 (0.40)
Logistic BN	22	0.55 (0.80)	0.50 (0.18)

Presented in the table are the number of predicted edges (p), true positive rate (TPR) and false discovery rate (FDR) for both directed and undirected (skeleton) edges using three different BN models. The true network is known and it contains 11 nodes and 20 edges.

### Simulation II: stepwise correlation-based feature selector

In the second simulation, we compare the proposed stepwise correlation-based feature selector with other existing methods. The feature selection step is to define a set of nodes of smallest possible size but include most possible nodes that are involved in the casual structure in the true phenotype-induced Bayesian network. Suppose we aim to select *p* variables from *S* (*S*>>*p*) candidates as the nodes in Bayesian network based on a random sample of *N* observations. Assume that the phenotype-induced Bayesian network truly has *p*+1 nodes (including the phenotype itself). The proposed stepwise feature selection method starts with the phenotype node and selects the features that are most correlated with the current nodes in a stepwise fashion based on a correlation or mutual information metric (in this paper, we use correlation). This procedure is a natural mimic of network structure and can identify those nodes indirectly associated with the phenotype. In practice, the method can be implemented as follows:

- Step 1: Calculate the correlation coefficients between the current node *X*_*i*_ and all the other nodes, denoted by *ρ*_*ij*_,*j*≠*i*. Keep *k* most correlated nodes with *X*_*i*_ based on *ρ*_*ij*_ for further filtering.
- Step 2: Calculate the p-value of correlation coefficient for each of the *k* nodes from step 1, select the node if the p-value is significant under Benjamini-Hochberg (BH) procedure with FDR ≤0.05.
- Step 3: Repeat step 1 and 2 until *p* nodes are selected.

In practice we need to pre-define the value of *p* and *k* based on the complexity of the network. The choice of *p* is subject to the feature pools size *S* and the scale of the network to build. The computing time is sub linear to *p*. We recommend to choose a *k* of 4, 5 or 6 to attain moderate complexity or sparsity of the network (see a simulation study below for the choice of k).

**Figure 12:**
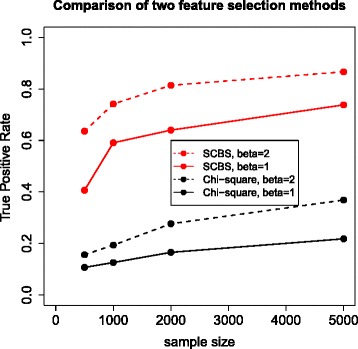
**Simulation II: Comparison between Pearson’s Chi-square test and SCBS procedure in feature selection.** Four curves presented in the plot are based on the true positive rate (TPR) by two methods under two different causal effects *β*=1 and *β*=2. Sample sizes are set to be 500, 1000, 2000 and 5000. The total number of positives is restricted to be 49 for both methods and the TPR is calculated as the number of true positives divided by 49.

To test the SCBS method, we conducted a simulation study with *S*=10,000 features, among which only *p*=49 are truly involved in the phenotype related network. We first generated 50 random sparse graphs (i.e., $\mathcal {G}=(V,E)$) consisting of 50 nodes and 100 directed edges (one node will be randomly chosen to be the phenotype node) using **bnlearn** ([40]). For each graph, we simulated eight samples according to the binomial logistic model, four with constant *β*_*ij*_=1 and the rest with *β*_*ij*_=2 at four different sample sizes *N*=500,1000,2000,5000. By using the topological order, each node can be simulated conditioning on the outcome of its parent nodes. For those nodes in the directed network but not caused by any other nodes, or the 9950 features outside the network, we simulated them from binomial distribution independently with success probability randomly generated from uniform distribution from 0.1 to 0.9. The SCBS with *k*=4 was applied to each data set and 49 features are identified from the sea of 10,000 candidates. Compared to the Pearson’s Chi-square test (single-round test for features between two phenotypic groups), the proposed SCBS performs uniformly better in all situations in terms of true positive rate (as shown in Figure 12, total positives were controlled at 49 for both methods). In particular, a small *β* poses more challenges in estimation or testing, while the SCBS can outperform the Chi-square test by 3-4 folds in true positive rate of identified phenotype-related features.

**Figure 13:**
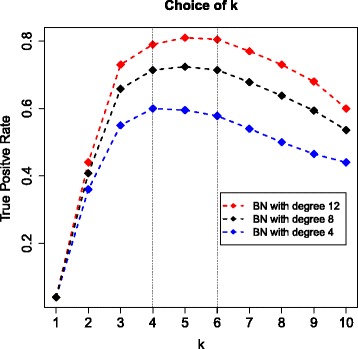
**True positive rate (TPR) of selected feature against the choice of** ***k*** **based on networks with average degree 4 (100 edges, blue curve), 8 (200 edges, black curve) and 12 (300 edges, red curve), respectively.** It is shown that increasing network density leads to increasing TPR, and a *k* of 4, 5 or 6 performs better in all situations.

A further simulation study on the choice of *k* was carried out using the same strategy as above with *S*=10,000, *p*=49, *β*_*ij*_=1 and *N*=1000. We generated 50 random graphs with average degree 4 (100 edges), 50 graphs with average degree 8 (200 edges) and 50 graphs with average degree 12 (300 edges), then apply SCBS with different *k* (*k*=1,2,…,10) to each data set and select 49 features. Figure 13 shows the true positive rates of the selected features under different choices of k for networks with different average degrees. It is shown that increasing network density leads to increasing true positive rates, and a *k* of 4, 5 or 6 performs better in all situations. Therefore for networks with moderate complexity, we recommend to choose *k*=4,5,6 since smaller *k* tends to miss weakly connected nodes and larger *k* tends to catch more false positives.

## Availability of supporting data

The data sets supporting the results of this article are available in the Cancer Genome Atlas repository, https://tcga-data.nci.nih.gov/tcga/.

## Additional files

Additional file 1
**Table S1.** Detailed information about the predicted network in Figure 6, including upstream (parent) and downstream (child) gene names for each gene in the network, gene locations (chromosome, strand, start and end positions), activation (represented by 1) or suppression (represented by -1) between two genes, affected by genetic/epigenetic changes or not.

Additional file 2
**Table S2.** Genes in each of the four clusters.

Additional file 3
**Table S3.** Detailed information about the survival-centric network in Figure 9, including upstream (parent) and downstream (child) gene names for each gene in the network, gene locations (chromosome, strand, start and end positions), activation (represented by 1) or suppression (represented by -1) between two genes, affected by genetic/epigenetic changes or not.

## Abbreviations

TCGA: The Cancer Genome Atlas
BN: Bayesian network
SCBS: Stepwise correlation-based feature selector
TPR: True positive rate
FDR: False discovery rate
